# Optimization of Dechlorination Experiment Design Using Lightweight Deep Learning Model

**DOI:** 10.1155/2022/1623462

**Published:** 2022-06-25

**Authors:** Jianghua Peng, Houzhang Tan

**Affiliations:** School of Energy and Power Engineering, Xi'an Jiaotong University, Xi'an 710049, China

## Abstract

This exploration intends to remove chloride ions in production and life, enhance buildings' durability, and protect the natural environment from pollution. The current dechlorination technology is discussed based on the relevant theories, such as the lightweight deep learning (DL) model and chloride ion characteristics. Next, data statistics and comparative analysis methods are used to study the adsorption and desorption performance of dechlorination adsorbents. Finally, the lightweight DL model is introduced into the chloride diffusion prediction experiment of slag powder and fly ash concrete. The results show that in the study of dechlorination adsorption performance, the chloride ion concentration decreases gradually with the extension of adsorption time. However, with the increasing temperature, the chloride ion removal rate is increasing. The removal rate of chloride ions in water can decrease slowly with the increase of adsorbent. Therefore, selecting the 2 mol/L sodium hydroxide as the alkali concentration for adsorbent regeneration is the most appropriate. Besides, the regeneration performance of the adsorbent gradually declines with the increase of sodium chloride concentration in the solution. The lightweight DL model is applied to the chloride diffusion prediction experiment of slag powder and fly ash concrete. It is found that when the curing age is selected at 18 days, 90 days, and 180 days, respectively, the error between the lightweight DL model and the experimental results is about 0.2. It shows that the lightweight DL model is feasible for predicting the diffusion of chloride ions. Therefore, this exploration designs and studies the dechlorination experiment based on the lightweight DL model, which provides a new theoretical basis and optimization direction for removing chloride ions in the future industry.

## 1. Introduction

With China's growing population, increased energy consumption, and intensified industrial activities, industrial wastewater treatment has become the focus of environmental protection [1]. The discharge of industrial wastewater will cause serious damage to the environment and cause great economic losses to enterprises. Excessive chloride ions in industrial wastewater have become a great challenge to industrial anticorrosion and attracted extensive attention, especially in the northwest region with relatively scarce water resources. Therefore, it is urgent to develop high-performance technology for chloride ion pollution control [2, 3].

Ma et al. studied the electroadsorption performance under different applied voltage and solution concentrations. It was found that the removal of sodium chloride increased with the increase of applied voltage and solution concentration, which was due to stronger electrostatic interaction, higher concentration gradient, and less double-layer overlap effect. Based on the Langmuir isotherm, the equilibrium electrosorption capacity at 1.2 V was determined to be 270.59 *μ*mol/g. In this case, due to the existence of micropores related to double-layer overlap, the effective surface area of ion electrosorption at 1.2 V was estimated to be in the range of 12.18–14.25% of the surface area of Brunauer–Emmett–Teller (BET) [4]. Leon-Fernandez et al. found that the chloride ion concentration higher than 100 mg·L^−1^ would affect the electrolytic zinc quality in the electrolytic zinc sulfate process. Hence, Engineering Capability Release (ECR) technology was used to treat chloride ions. The electrode adopted two copper electrodes with an area of 3.15 cm^2^ and an Ag/AgCl reference electrode, and the distance between the two copper electrodes was 5 mm. Under the optimal conditions of voltage 0.6 V, ultrasonic power 50 W, and reaction time 3 h, the chloride ion removal rate reached 54.5% when the chloride ion concentration was 300 mg·L^−1^ [5]. Gong et al. used a genetic algorithm and lightweight deep learning (DL) model to optimize the experiment of the dechlorination training set. The results of this machine learning model were very accurate. However, this algorithm also has certain limitations. It usually requires massive training data to achieve a certain accuracy, and it will still show great error in predicting the molecular properties outside the sample [6]. Hubacek et al. proposed applying a lightweight DL algorithm to predict the total molecular energy of chemical structures with different spatial coordinates and charges, including organic molecules, inorganic molecules, and ions [7].

To sum up, this exploration first expounds on the commonly used dechlorination technology and then studies the adsorption and desorption performance of dechlorination adsorbents. Furthermore, the lightweight DL model is applied to the chloride diffusion prediction experiment of slag powder and fly ash concrete. The research contribution is to improve and optimize the existing dechlorination experiments and design a lightweight DL prediction method with high efficiency, speed, and accuracy. This exploration provides more methodological references for industrial dechlorination in the future and enriches the research theory in this field.

## 2. Materials and Methods

### 2.1. Lightweight DL Model

**Figure 1:**
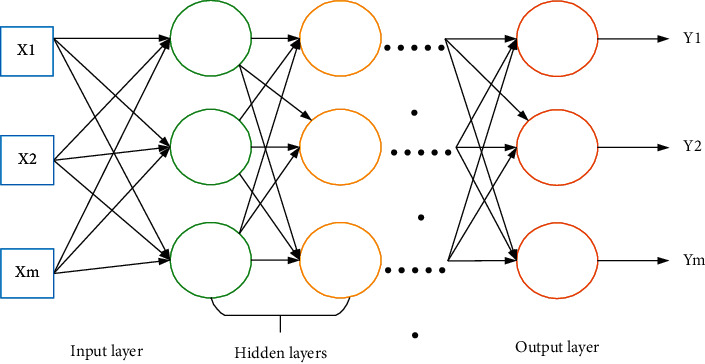
DL model (*X* represents input data and *Y* represents output data).

DL is to learn the sample data's internal law and representation level. The information obtained in the learning process is beneficial to interpreting data such as text, images, and sound [8, 9]. Its ultimate goal is to make the machine have the ability to analyze and learn like human beings and recognize characters, images, sounds, and other data. DL is a complex machine learning algorithm that has achieved far more speech and image recognition results than previous related technologies [10].  Figure 1 shows the specific model structure.

Classified from the specific research content, DL mainly involves three kinds of methods: convolutional neural network (CNN) model, self-coding neural network of multilayer neurons, and deep confidence network [11].

**Figure 2:**
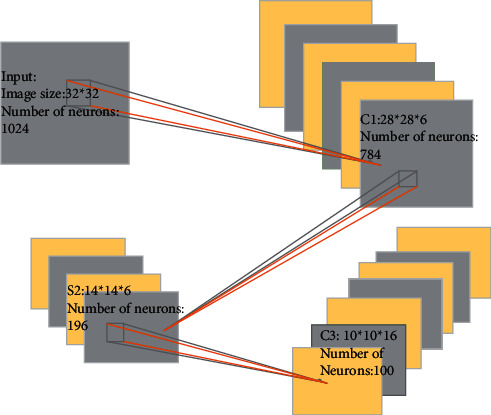
CNN model.

**Figure 3:**
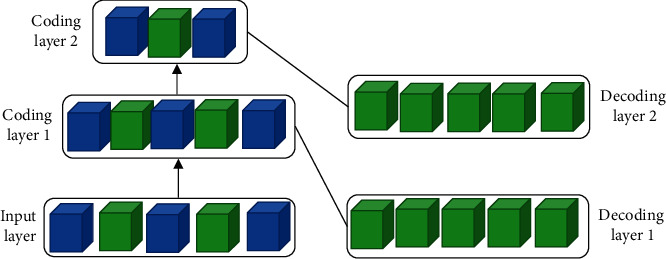
Self-coding neural network model (the blue cube represents the input quantity and the green cube represents the output quantity).

(1) CNN is a kind of feedforward neural network with convolution calculation and depth structure. It is one of the representative algorithms of DL. CNN has the ability of representational learning and can conduct the translation-invariant classification for the input information according to its hierarchical structure. Therefore, it is also called a “translation-invariant artificial neural network,” mainly composed of input, hidden, and output layers.  Figure 2 shows the specific form.
If *x*_1_, *x*_2_, *x*_3_,…, *x*_*n*_ are input signals and *n* neurons are connected to each other, the first neuron is used as an object, which inputs information into all other neurons *j*(*j*=1,2,…, *n*). The connection weight from the *j*th neuron to the *i*th neuron is expressed as *ω*_*ij*_. With the quasi-linear element model in the neuron model as an example, the biggest feature of the model is to use continuous information as input and output. The following equation is the calculation method of the output function *f*(*x*):
  (1) $$\begin{array}{c} f\left(x\right)=\frac{1}{1+\text{exp}\left(-x+\theta_{i}\right)}. \end{array}$$
The total input *μ*_*i*_ of neurons in the model is expressed as
  (2) $$\begin{array}{c} \mu_{i}=\sum_{j=1}^{n}\omega_{\text{ij}}x_{j}-\theta_{i}, \end{array}$$
  where the output of neuron *i* can be calculated by substituting *μ*_*i*_ into the following equation as variable *x*.
  (3) $$\begin{array}{c} y_{i}=f_{i}\left(\mu_{i}\right). \end{array}$$
The output value *y*_*i*_ is a continuous value.
Besides, when the data in the input layer are *x*_1_, *x*_2_, *x*_3_,…, *x*_*n*_, the input of each neuron in the hidden layer is as follows:
  (4) $$\begin{array}{c} h_{i}=\sum_{j=1}^{n}z_{\text{ij}}x_{j}+p_{i}\left(i=1,2,\ldots ,\tau \right), \end{array}$$
  where *n* and *τ* are the number of neurons in the input layer and hidden layer, respectively. *z*_*ij*_ is the connection weight between *j* neuron in the input layer and *i* neuron in the hidden layer; *p*_*i*_ is the threshold value of *i* neurons in the hidden layer; *h*_*i*_ is the input value of *i* neuron in the hidden layer [12].
The activation function of neurons in the hidden layer adopts the sigmoid function, so the expression of the output of neurons in the hidden layer reads:
  (5) $$\begin{array}{c} o_{i}=f\left(h_{i}\right)\left(i=1,2,\ldots ,\tau \right). \end{array}$$
Activate function is *f*(*x*)=1/1+*e*^−*x*^. The activation function of neurons in the output layer adopts the identity function. If the threshold value is 0, the output of each neuron in the output layer can be expressed as follows:
  (6) $$\begin{array}{c} y_{k}=\sum_{i=1}^{\tau }v_{ki}o_{i}\left(k=1,2,\ldots ,l\right), \end{array}$$
  *v*_*ki*_ is the connection weight between *i* neurons in the hidden layer and *k* neurons in the output layer; *l* is the number of neurons in the output layer.
The connection weights between the input layer and hidden layer neurons together form the weight vector *Q*. After the weight vector *Q* is determined, the output value can be calculated according to the input value of the neural network. The value of the weight vector *Q* is random in the initial case, so the actual output *y*_*kr*_ accuracy of the calculated network is not high. After determining the number of neurons in the hidden layer *τ*, the error *d*_*kr*_ [13] can be reduced by adjusting the *Q* value. Back propagation is along function *e*_*r*_. The weight vector is adjusted with the negative gradient direction of the weight vector. The correction value of the weight vector *Q* is set as Δ*Q*, Δ*Q*=−*η∂e*_*r*_/*∂Q*. *s* is the learning rate, and the value range is 0∼1. The following equations are obtained through calculation:
  (7) $$\begin{array}{c} \Delta Q=\eta \sum_{k=1}^{l}d_{\text{kr}}\frac{\partial y_{\text{kr}}}{\partial Q}. \end{array}$$
  (8) $$\begin{array}{c} \Delta Q=\left(\Delta v_{\text{ki}},\Delta p_{i},\Delta Q_{\text{ij}}\right). \end{array}$$
  (9) $$\begin{array}{c} \Delta v_{\text{ki}}=\eta d_{\text{kr}}o_{\text{ir}}. \end{array}$$
  (10) $$\begin{array}{c} \Delta p_{i}=\eta o_{\text{ir}}\left(1-o_{\text{ir}}\right)\sum_{k=1}^{l}d_{\text{kr}}v_{\text{ki}}. \end{array}$$
  (11) $$\begin{array}{c} \Delta Q=\eta o_{\text{ir}}\left(1-o_{\text{ir}}\right)x_{\text{jr}}\sum_{k=1}^{l}d_{\text{kr}}v_{\text{ki}}. \end{array}$$
The value of Δ*Q* can be calculated by the above equation, and then the weight vector can be corrected by *Q*=*Q*+Δ*Q* to obtain the corrected weight vector [14].
(2) Self-coding neural networks based on multilayer neurons include Autoencoder and Sparse Coding, which have recently attracted extensive attention.  Figure 3 shows the specific model.
The specific calculation method is expressed as
  (12) $$\begin{array}{c} \min\sum_{m}^{M}L\left(A^{m},B^{m},C^{m}\right)+\lambda \text{ Reg}\left(C\right), \end{array}$$
  where *A*^*m*^,  *B*^*m*^,  and *C*^*m*^ represent the input *A*,  *B*,  and *C* matrices of the *m*th task, respectively; *M* is the total number of samples; Reg represents a regularized constraint; and *λ* is the weight that controls the regularization constraint [15].
(3) Deep belief network (DBN) is a kind of neural network of machine learning, which can be used for both unsupervised learning and supervised learning. DBN is a probability generation model. Compared with the neural network of the traditional discrimination model, the generation model is to establish a joint distribution between observation data and labels. The whole neural network can generate training data according to the maximum probability by training the weights between its neurons [16].

DL algorithm has achieved great success in the field of computer vision, such as image classification and target detection. However, due to storage space and computation limitations, DL technology application in embedded and mobile devices with certain storage and computing requirements is still a great challenge. How to compress the model to reduce storage space and computing consumption has become a research hotspot. Present compression is mainly realized through the two methods below [17, 18].

#### 2.1.1. Matrix Decomposition Algorithm

Matrix decomposition is a commonly used algorithm in model compression, including singular value decomposition (SVD) or low-rank decomposition algorithm. The idea is to obtain the most representative information in the parameter matrix of each layer by approximate estimation to realize the effect of accelerating the operation speed of the compression model [19]. For example, if the number of fully connected input layers is *x*, the size is *u* × *v*, and the weight matrix is *w*, the calculation method of the output data *y* of the fully connected layer is as follows:

(13) $$\begin{array}{c} y=Wx. \end{array}$$

If the singular value decomposition is performed on *W* and the first *t* important eigenvalues after decomposition are approximately used to replace *W*, the following equation displays the decomposition calculation method:

(14) $$\begin{array}{c} W=U\sum V^{T}\approx U\sum_{t}V_{r}^{T}, \end{array}$$

where *U* represents an orthogonal matrix in *u* × *t* dimension, Σ_*t*_ is a diagonal matrix corresponding to the first *t* values in the original diagonal matrix *W*, and *V* represents an orthogonal matrix in *v* × *t* dimension. From the above equation, the following equation displays the specific calculation method of singular value decomposition:

(15) $$\begin{array}{c} y=\text{Wx}\approx U\cdot \left(\sum_{r}V^{T}\right)\cdot x=U\cdot z. \end{array}$$

#### 2.1.2. Modify the Original Network Structure

**Figure 4:**
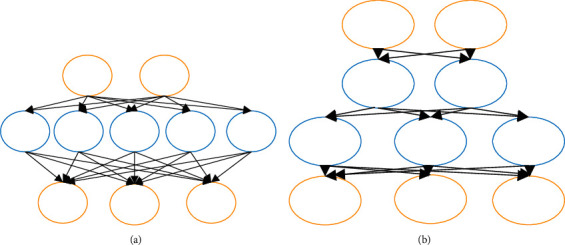
Compression decomposition of neural network ((a) the network with large width and small depth; (b) the network with small width and large depth).

Replacing network width with network depth is one of the methods to realize network model compression.  Figure 4 shows the specific form.

Figure 4(a) is characterized by the neural network's large width and small depth.  Figure 4(b) is characterized by the neural network's small width and large depth. The advantage of increasing the depth is that the more parameters the neural network carries, the more data it can train at one time. Then, it is more favorable for the test data inspection and analysis [20, 21].

**Figure 5:**
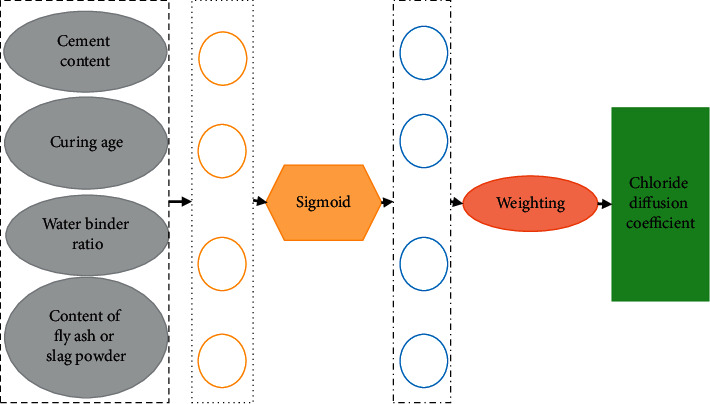
Teaching design flow of dechlorination experiment based on lightweight DL.

The lightweight DL model is introduced into the design and research of the dechlorination experiment to enhance the accuracy and convenience of the experiment.  Figure 5 shows the specific design process.

Figure 5 reveals that factors such as cement content, fly ash content (or slag powder), and curing time are selected as input and chloride ion diffusion coefficient as output. The autonomous learning ability of the CNN is adopted to train the prediction model. In data processing, *P*_max_ is equal to 270 and 2.2 × 10^−11 ^m^2^/s, the maximum value of input and output values, respectively. Four 2 × 2 parameter convolution kernels are set in the convolution feature layer, and the sigmoid function is adopted as the activation function.

### 2.2. Characteristics of Chloride Ion and Dechlorination Technology

Chlorine (Cl) is a nonmetallic element. It is a yellow-green gas under normal temperature and pressure; has a strong pungent smell and very active chemical properties; and is toxic [22, 23]. Chlorine widely exists in nature in the compound form, which is also of great significance to human physiological activities [24]. Sodium chloride salt is common in life.

The following equation is the calculation method of one-dimensional chloride diffusion in concrete:

(16) $$\begin{array}{c} \frac{\partial C\left(x,t\right)}{\partial t}=\frac{\partial }{\partial t}\left(D\frac{\partial C\left(x,t\right)}{\partial x}\right), \end{array}$$

where *C*(*x*, *t*) represents the chloride ion content at *x* away from the erosion surface when the erosion time is *t*, and *D* represents the effective chloride ion diffusion coefficient.

At present, there are mainly three kinds of dechlorination technologies, namely electrochemical removal method, physicochemical method, and chemical method. (1) Electrochemical method is a method to transfer or transform chloride ions in solution through an external current and finally realize the separation of chloride ions from the solution to be treated. Different from other methods, electrochemical treatment has a high degree of automation and does not need additional agents. However, the energy consumption of electrochemical treatment is large, and it is difficult to produce concentrated liquid or chlorine gas. In general, electrochemistry has a good application prospect in circulating water treatment. The overall operation of the electrochemical method is relatively simple and clean without adding chemical reagents. Electrochemical energy is relatively popular and easy to obtain. However, the relative energy consumption is large and the treatment cost is high [25]. (2) Physicochemical methods are to remove chloride ions through interception or adsorption technology, including the methods of reverse osmosis and ion exchange resin treatment. Reverse osmosis technology is a membrane treatment technology with high removal efficiency and can reduce chloride ions to a small amount. However, there are membrane damage and high energy consumption. At present, many studies are about membrane modification. Membrane modification can reduce membrane damage and increase membrane permeability. The ion exchange resin method refers to the replacement of the exchangeable group on the resin with the chloride ion to be removed from the electrolyte. The treatment efficiency of the ion exchange method is high, and the resin can be reused. However, the resin has incomplete desorption, and its adsorption and desorption properties are changed by changing the properties of the resin [26, 27]. (3) Chemical dechlorination methods mainly include the ultra-high lime aluminum method, layered bimetallic hydroxide, copper powder precipitation, and blowing in NO_2_. First, the principle of the ultra-high lime aluminum method is to react with Cl- in the presence of Ca(OH)_2_ and Al(OH)_3_ to form Ca_4_Al_2_(OH)1_2_Cl_2_. The ultra-high lime aluminum process has sufficient raw materials and low requirements for water quality and impact load resistance. However, its raw material utilization rate is low, the reaction conditions are complex, and the effluent hardness will increase [28]. Layered bimetallic hydroxide is a kind of anionic clay with a layered structure. The calcined products of bimetallic hydroxides can reabsorb water and anions under certain conditions by using the structural memory effect to partially restore the layered structure of lactate dehydrogenase [29]. Layered double-layer hydroxide has a stable effect and high chloride ion removal efficiency, but chloride ions will be affected by the presence of other anions [30, 31]. Principle of copper powder dechlorination: copper and copper ions interact with chloride ions in solution to form cuprous chloride precipitation. Copper powder precipitation may introduce new impurities and the high cost of chlorine remover. However, this method can be used in mining and metallurgy industries with low chlorine concentrations to realize waste utilization [32, 33].

### 2.3. Experimental Equipment and Methods

Tables 1 and 2 show the drugs, reagents, instruments, and equipment used.

## 3. Results and Analysis

### 3.1. Study on Adsorption and Desorption Properties of Dechlorination Adsorbent

**Figure 6:**
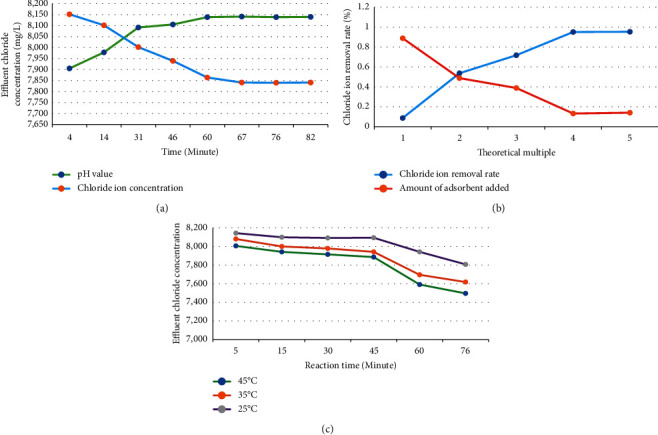
Adsorption performance of dechlorination adsorbent (Figure (a) shows the effect of reaction time on the dechlorination of adsorbent. Figure (b) shows the effect of adsorbent addition on the dechlorination of adsorbent. Figure (c) shows the effect of adsorption temperature on the dechlorination of adsorbent).

Based on the above experimental conditions, the adsorption performance of dechlorination adsorbent is studied.  Figure 6 shows the specific results.

Figure 6 suggests that the chloride ion concentration gradually decreases with the extension of adsorption time. After 65 minutes, the chloride ion concentration does not change, indicating that the maximum adsorption capacity has been reached. Meanwhile, the pH will gradually rise during the reaction. Therefore, at the beginning of the reaction, a certain amount of sulfuric acid needs to be added to maintain the subsequent consumption of sulfuric acid. The removal rate of chloride ions in water can decrease almost in proportion with the addition of adsorbent.  Figure 6(b) shows the results, suggesting that the chloride ion dosage can control the chloride ion removal rate, and the maximum chloride ion removal rate can reach more than 98.01%.  Figure 6(c) reveals the effect of temperature on the adsorption performance of the material. It reveals that the influence of temperature on the dechlorination process is quite obvious. With the increase of temperature, the removal rate of chloride ions increases.

**Figure 7:**
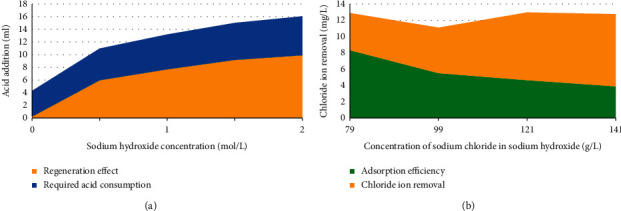
Desorption performance of dechlorination adsorbent. (Figure (a) shows the effect of NaOH concentration on adsorbent regeneration performance; Figure (b) reveals the effect of NaCl concentration in regeneration solution on adsorbent dechlorination).

Figure 7 is the research result of adsorbent desorption performance.

Figure 7(a) shows that the regeneration effect will be improved when the alkali concentration increases. However, the acid consumption required for subsequent neutralization and the cost of dechlorination will increase. The adsorbent's regeneration effect is poor using a low alkali concentration. 2 mol/L sodium hydroxide is selected as the alkali concentration for adsorbent regeneration through comprehensive comparison.  Figure 7(b) shows that the regeneration performance of the adsorbent decreases gradually with the increase of NaCl concentration in the solution. When the NaCl concentration in the regeneration solution reaches 120 g/L, the adsorbent can still maintain 60% adsorption efficiency after regeneration. Upon conversion, this is equivalent to the salt concentration of alkali regeneration solution after three times of continuous neutralization. Therefore, the same batch of regeneration solution can be used for three times during the experiment. In addition, when the calcium hardness in the regeneration solution is less than 500 mg/L, it has little effect on the regeneration performance of the regeneration solution. The follow-up study reveals that even if the raw water hardness (CaCO_3_) reaches 11310 mg/L, the adsorbed adsorbent enters the alkali regeneration solution after 0.1 mol/l sulfuric acid pickling and twice water washing, and the hardness was less than 500 mg/L, so it has little impact on the subsequent alkali reuse.

### 3.2. Study on the Optimization of Dechlorination Experimental Design of Lightweight DL Model

**Figure 8:**
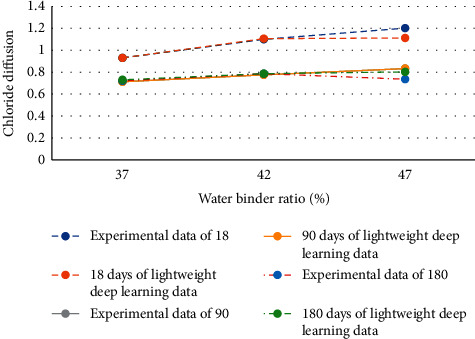
Prediction results of lightweight DL model in chloride diffusion of slag powder concrete.

Seaports and sea crossing bridges are often eroded by chloride ions, resulting in reinforcement corrosion and concrete surface falling off. Therefore, the prediction results of the lightweight DL model in chloride diffusion of slag powder concrete are analyzed (Figure 8).

Figure 8 displays that the curing age of concrete is selected as 18 days, 90 days, and 180 days, respectively. Moreover, the experimental data are compared with the experimental data added with the lightweight DL algorithm. When the curing age is 18 and the water to binder ratio is about 41%, the predicted value of the lightweight DL model deviates from the experimental value. When the curing age is 90 days, the values completely coincide. When the curing age is 180 days, the data coincide only when the water to binder ratio is about 41%. It reveals that when the curing age is 90 days, the prediction result data of the lightweight DL model are completely consistent. However, the figure also reveals that the error values are within 0.2, indicating that the values predicted by the lightweight DL model show a strong correlation with the chloride diffusion value in the experiment.

**Figure 9:**
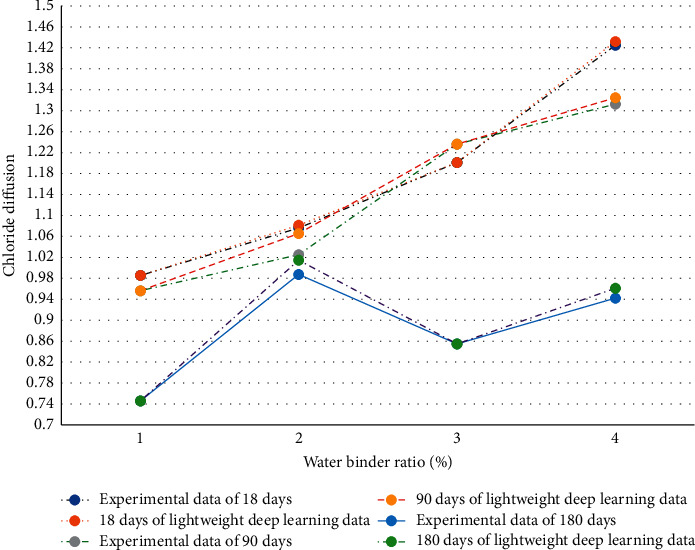
Prediction results of lightweight DL model in chloride diffusion of fly ash concrete.

Moreover, the prediction results of the lightweight DL model in chloride diffusion of fly ash concrete are analyzed (Figure 9).

Similar to Figure 8, the experimental data are compared with the experimental data added with the lightweight DL algorithm. Figure 9 shows that there are errors to a certain extent in the curing age of 18 days, 90 days, and 180 days, among which the largest error is the chloride ion prediction result of the fly ash concrete in 90 days. However, similar to the prediction results of chloride diffusion in slag powder concrete, although there are errors, they are all in the range of 0.2. Therefore, it can be preliminarily determined that the lightweight DL model has certain applicability to the prediction results for chloride ion diffusion.

## 4. Conclusion

With the continuous progress of industrialization, soil, water quality, and air have also been polluted, and there are multiple kinds of pollution, among which the excessive concentration of chloride ions is one of them. First, the adsorption and desorption properties of the dechlorination adsorbent are analyzed. Then, the lightweight DL model is introduced into the chloride diffusion prediction experiment of slag powder and fly ash concrete. The data statistics and comparative analysis methods are used to draw the following conclusions: (1) After studying the adsorption performance of dechlorination adsorbent, it is found that the chloride ion concentration decreases gradually with the extension of adsorption time. The removal rate of chloride ions in water can decrease almost in proportion to the addition of adsorbent. With the increase of temperature, the removal rate of chloride ions will also increase. (2) The desorption performance of the dechlorination adsorbent is analyzed. It is found that when the alkali concentration increases, the regeneration effect will be improved. However, the acid consumption required for subsequent neutralization and the dechlorination cost will increase. However, the regeneration effect of the adsorbent is poor using a low concentration of alkali. 2 mol/L sodium hydroxide is selected as the alkali concentration for adsorbent regeneration after a comprehensive comparison. With the increase of NaCl concentration in the solution, the regeneration performance of the adsorbent decreases gradually. (3) The prediction results of the lightweight DL model in chloride diffusion of slag powder concrete are analyzed. Thereafter, it is found that when the curing age of slag powder and fly ash is selected at 18 days, 90 days, and 180 days, respectively, there will be some errors more or less, but they are all in the range of 0.2, indicating that the lightweight DL model has certain applicability to the prediction results of chloride diffusion.

Due to the limited energy, there are some limitations in data acquisition, resulting in some deviations in the inspection of relevant data. There is also a study on the design of a dechlorination experiment based on a lightweight DL model, which has not been discussed regarding economic cost investment. According to the specific situation, the benefit evaluation can be carried out in the follow-up research. In this way, to a certain extent, it can bring certain methods and optimization directions for the dechlorination experiment in production and life. [34].

## Figures and Tables

**Table 1:** Drugs and reagents used in the experiment.

Reagent name	Chemical equation	Purity	Production company
Isopropyl titanate	Ti{OCH(CH_3_)_2_}_4_	Chemically pure	A company in Zhejiang

Sulfuric acid	H_2_SO_4_	Analytical pure	Chinese medicine reagent
Nitric acid	HNO_3_		
Hydrochloric acid	HCl		
Sodium chloride	NaCl		
Silver nitrate	AgNO_3_		
Potassium chromate	K_2_CrO_4_		Aladdin reagent
Sodium hydroxide	NaOH		Chinese medicine reagent
Calcium sulfate	CaSO_4_		
Magnesium sulfate	MgSO_4_		

Deionized water	H_2_O	*R* > 10 Ω·cm	Laboratory self-made

**Table 2:** Experimental equipment.

Name of instrument and equipment	Type	Manufacturer
Ultra-pure water system	EPED-40TF	Nanjing Yipu Yida Science and Technology Development Co.
Magnetic stirrer	Topolino	IKA, Germany
Analytical balance	AL104	METTLER TOLEDO
Centrifuge	L500	Xiangyi centrifuge instrument co., ltd.
Electric blast drying oven	DHG-9245A	Shanghai Yiheng Scientific Instrument Co., ltd.
Intelligent thermostatic bath	DC-2006	Ningbo Xinzhi Biotechnology Co., ltd.
Circulating water vacuum pump	SHB-III	Changsha Mingjie Instrument Co., ltd.
Constant temperature shaking table	ZWY-2000	Shanghai Zhicheng
pH meter	PHS-3C	Shanghai leici Technology Co., ltd.
Pipettor	Finnpipette F2	Shanghai Chuangyi Science and Education Equipment Co., ltd.
Constant flow peristaltic pump	BT100MH	Baoding Chuang Rui Precision Pump Co., ltd.

## Data Availability

The data used to support the findings of this study are included within the article.
